# It is unprecedented: trial management during the COVID-19 pandemic and beyond

**DOI:** 10.1186/s13063-020-04711-6

**Published:** 2020-09-11

**Authors:** Eleanor J. Mitchell, Khaled Ahmed, Suzanne Breeman, Seonaidh Cotton, Lynda Constable, Gillian Ferry, Kirsteen Goodman, Helen Hickey, Garry Meakin, Katy Mironov, Niamh Quann, Natalie Wakefield, Alison McDonald

**Affiliations:** 1 Nottingham Clinical Trials Unit, University of Nottingham, Building 42, University Park, Nottingham, NG7 2RD UK; 2 Birmingham Clinical Trials Unit (BCTU), Institute of Applied Health Research, College of Medical and Dental Sciences, Public Health Building, University of Birmingham, Edgbaston, Birmingham, B15 2TT UK; 3 Centre for Healthcare Randomised Trials (CHaRT), University of Aberdeen, Health Sciences Building, Foresterhill, Aberdeen, AB25 2ZD UK; 4 NMAHP Research Unit, Glasgow Caledonian University, Cowcaddens Road, Glasgow, G4 0BA UK; 5 Liverpool Clinical Trials Centre, University of Liverpool, a member of the Liverpool Health Partners, Liverpool, L69 3BX UK; 6 Oxford Trauma, University of Oxford, NDORMS, Kadoorie Centre, Level 3, John Radcliffe Hospital, Oxford, OX3 9DU UK; 7 Leicester Clinical Trials Unit, College of Life Sciences, University of Leicester, University Road, Leicester, LE1 7RH UK

**Keywords:** COVID-19, Pandemic, Clinical trial, Trial management

## Abstract

The COVID-19 pandemic has presented unique challenges for the clinical trial community, both in the rapid establishment of COVID-19 clinical trials and many existing non-COVID-19 studies either being temporarily paused (whether that is a complete pause or pause in some activities) and/or adapting their processes. Trial managers have played a key role in decision-making, undertaking risk assessments and adapting trial processes, working closely with other members of the research team. This article presents some of the ways in which trial management processes have been altered and the key role that trial managers have played. It has been born out of discussions between trial managers in the UK who are members of the UK Trial Managers’ Network (UKTMN), a national network of trial management professionals managing non-commercial trials.

In these unprecedented times, clinical trials have faced many uncertainties and broad-ranging challenges encompassing a range of activities including prioritising patient safety amidst the pandemic, consenting and recruiting new participants into trials, data collection and management and intervention delivery. In many cases, recruitment has been paused whilst mitigations have been put in place to continue data collection. Innovative solutions have been implemented to ensure we continue, where possible, to deliver high-quality clinical trials. Technology has provided many solutions to these challenges, and trial managers have adapted to new ways of working whilst continuing to deliver their clinical trials. Trial management groups are now faced with new uncertainties around re-starting clinical trials, and it is unclear currently how this will go, though working together with sponsors, funders and site teams is clearly a priority.

Clinical trial teams have worked together to ensure their trials have adapted quickly whilst ensuring participant safety is given utmost importance. There are clear examples where the trial community have come together to share experiences and expertise, and this should continue in the future to ensure the innovative practices developed become embedded in the design and conduct of clinical trials in the future.

## Background

On 11 March 2020, the World Health Organization (WHO) declared the outbreak of the COVID-19 pandemic [1]. Across the globe, countries implemented a variety of restrictions to people’s everyday lives which have included, country-wide lockdowns, social distancing and, for many where it is possible to do so, working from home. The impacts on clinical research have been significant. Many researchers around the world have acted quickly, designing and conducting new clinical trials and research studies to obtain valuable evidence on the virus and impact upon national and international healthcare policies. Upon checking the UK’s Health Research Authority (HRA) website for ethically approved COVID-19 research on 4 June 2020, 142 studies had been approved since 24 February 2020 [2], of which 49 have been classified as an urgent public health COVID-19 study [3]. All of this has been done whilst many people adapted to working from home whilst, in many cases, juggling caring and home schooling responsibilities. In addition to the rapid establishment of studies investigating the virus itself, there has been a significant impact upon existing research studies and clinical trials. In the UK, the largest funder of clinical research, the National Institute for Health Research (NIHR), issued guidance enabling clinical and academic healthcare professionals who were working on existing non-COVID studies within the NIHR portfolio, to support the NHS by returning to clinical care duties [4]. In addition, the Clinical Research Network (CRN) paused set-up of any new or ongoing studies at sites, unless a national priority for urgent public health research into COVID-19 [5]. Other agencies such as the European Medicines Agency (EMA) also issued guidance on the management of clinical trials during the pandemic [6]. This led to research teams and sponsors urgently revising risk assessments to consider the implications and impact on their clinical trials. Following the release of these recommendations, trial management groups (TMGs) were tasked with considering the implications upon pausing some or all aspects of studies, particularly if such a pause could impact upon the care or safety of trial participants. Trial management staff, working closely with Chief Investigators and other members of the immediate research team, have been at the heart of leading discussions about the impact of COVID-19 on their trials.

The UK Trial Managers’ Network (UKTMN) is a national network of around 800 trial management professionals, responsible for the day-to-day running of clinical trials and other large research studies. Through discussions in the UKTMN executive group, UKTMN membership via social media and online networking events and wider experiences of trial managers amongst some Clinical Trials Units (CTUs), this article presents some of the challenges trials have faced and discusses some of the innovative solutions that have been implemented in order for trials to continue in the future, beyond the pandemic. We specifically focus on the impact to non-COVID-19 studies, rather than studies that have been designed to focus on COVID-19 itself. At the time of writing, we are not aware of other articles that consider the implications on trial management during the COVID-19 pandemic.

## Problems and solutions

### Trial recruitment and consent

Many trials have been paused to recruitment due to lack of site research staff and the need to minimise face-to-face contact with participants and potential participants [4]. Some studies which have continued have seen a significant reduction in their recruitment figures. A hybrid model has been adopted in some cases, in keeping with a proportionate approach to risk assessment, ensuring each study is considered on an individual basis. One example of this is a trial whereby the sponsor approved recruitment to continue in circumstances where potential participants had already been approached for participation, but had not yet provided postal consent, a process which was already embedded within the consent pathway [7]. A further example is of a trial conducted in the Emergency Department (ED), where after a patient’s initial attendance in ED, all subsequent activities were conducted online [8]. Other ways of communicating with potential participants are also being explored; a trial in patients with aortic stenosis is currently considering plans to implement a process for using video-conferencing with patients in order to undertake the consent process remotely [9].

Obtaining informed consent for participation in a clinical trial is a regulatory and ICH-GCP (Good Clinical Practice) requirement [10]; this is commonly performed in person where participants are asked to complete and sign a paper consent form. However, whilst the use of electronic informed consent is increasing, in our experience, it is still relatively uncommon. During this pandemic, some trials have utilised electronic consent methods in order to continue trial recruitment whilst minimising face-to-face contact with potential participants. These systems have been set-up quickly, often with support from specialist programmers. In some circumstances, they have been used as an alternative to paper consent, though still used in the context of a face-to-face setting. Whilst this reduces the amount of paper handling and arguably provides a more streamlined efficient approach to consent in trials, electronic consent could be most useful when truly “online” and not reliant upon site staff providing participants with a smart device to complete the electronic consent form. We anticipate that more trials in the future will develop electronic consent systems, though careful consideration needs to be given on a trial-by-trial basis particularly considering the demographics of the study population, ensuring a proportionate approach, and adherence with guidance provided by the HRA and Medicines for Healthcare Regulatory Authority (MHRA) [11]. Using consistent methods to record an electronic signature and ensuring research staff gain experience in taking consent in this way will be important.

### Communication with site staff and other stakeholders

Regular communication with site staff is a key aspect of trial management [12, 13]. Though recruitment in many trials is paused, communication continues to be crucial. For TMGs to make ongoing decisions about changes to a trial, whether that be pausing/re-starting or a change in trial process, input from site staff before decisions are finalised is vital. Principal Investigators and research nurses at sites offer valuable perspectives into what is happening “on the ground” and their absence in decision-making could result in unintended consequences for the trial. Their input into approaching trials holistically is crucial since to consider one element of a trial alone, e.g. recruitment or retention, could have a negative impact upon other areas of the trial. Trial managers have been required to test communication styles with sites, working with sites to ensure they use the best communication method for them (which may differ between sites). Additionally, it has been necessary in some trials to ascertain which site staff, if any, have the capacity to undertake any trial-related activity. This has ensured that the right communication is targeted to the right people at the right time [14, 15].

In addition to communicating with site staff, trial managers are also in close communication with trial funders and sponsors. Some trial managers based in CTUs, who are working on multiple trials, report that a significant challenge has been communicating with multiple sponsors, all of whom may interpret guidance differently and stipulate different requirements for risk assessment, reporting and considering restarting trials. Others have mitigated this by proposing processes directly to sponsors. A substantial amount of time has been spent by many trial managers in completing documentation, such as risk-assessments, contingency plans, questionnaires and reports for a variety of stakeholders, e.g. funders, sponsors, Trial Steering Committees (TSCs) and Data Monitoring Committees (DMCs). A streamlined approach to reporting would be welcomed and allow trial managers to focus their time on other trial-related activities.

### Intervention delivery

Clinical trial interventions are broad-ranging, and a key aspect of adapting the trial risk assessment during the pandemic has been whether the intervention can still be delivered to participants safely. It has been paramount that TMGs have carefully considered the safest option(s) for intervention delivery; in some cases, it may have been safer to stop the intervention entirely, and in others, finding a mechanism to deliver the intervention safely has been implemented. For example, in some trials, it has been deemed safe to deliver study medication directly to the participant, without the requirement for a face-to-face visit [16]. In other trials, trial managers report participants stopping trial medications due to COVID-19 concerns; however, many were prepared to continue with remote follow-up. Other types of interventions may be delivered alongside routine clinical practice, for example, surgical interventions. Since much routine surgery has been postponed due to the pandemic, this has clearly impacted upon participants being unable to have their trial intervention and thus overall trial timelines. Trials that have a qualitative component or where the intervention is, for example, a therapy session (individual or group-based) may be able to be conducted remotely via video-conferencing.

### Data collection

Though in many cases trial recruitment has either paused or reduced considerably, in some trials, it has been possible to continue to collect data (for those already recruited into the trial or for those trials who have completed recruitment and are now in follow-up). In some instances, where data collection is largely based upon utilising data from existing clinical records, some processes have been unaltered, though there has been an impact upon data entry timelines. For some trials, data entry and data query resolution is being undertaken by trial managers, though this has presented logistical challenges whilst working at home. In other trials where data is largely participant-reported, questionnaire completion has shifted from postal questionnaires to telephone or online data collection. Each approach has challenges: for example, the time taken to collect data by telephone and potential issues in collecting sensitive and/or clinical information over the telephone. Converting to online data collection may involve additional programming time to build the functionality into a trial database and will require participants to have provided an email address or mobile phone number. These changes have perhaps been easier to implement in departments where online data collection had been used or explored previously, but could be challenging and not necessarily a “quick fix” when this option had previously not been explored before. Attention has also been given to collecting data from other sources, such as biological samples. For example, a trial investigating incentives for smoking cessation in pregnancy had been designed to collect saliva samples at face-to-face clinic visits; a substantial amendment has been approved in order to send the sample kits directly to the participant’s home along with an envelope to return this [17].

Trial managers reported undertaking a risk assessment when considering data collection, many focussing only on collection of critical data such as primary outcome and safety data. The implications of any secondary data not collected should be agreed within the TMG and discussed with oversight committees. In other circumstances, some trials in set-up are adding in additional data items to their data collection tools to collect clinical data relating to COVID-19, though it is recognised this should be done in a streamlined way, utilising a core outcome set where possible. Although only data that is used for outcomes should be collected, we recognise this is not always the case, with additional non-critical data commonly being collected. This has been confirmed by the recently published DataCat project which concluded that a substantial amount of data collection is not related to trial outcomes [18]. In trials that have altered their data collection processes and methods, initial feedback suggests participants have responded favourably, though in many cases it is too early to formally evaluate the impact upon participant retention. However, this may present an opportunity for methodological work to assess the impact of revised data collection pathways and, for example, shorter questionnaires.

In addition to considerations from a data collection perspective, the impact upon analysis of trial data is important to consider. It has been crucial that trial managers have worked closely with trial statisticians and health economists, in addition to other members of the TMG, whilst considering this. For example, analysis plans may need to be adapted to accommodate changes such as timing of data collection and the way in which data is being collected, alongside the wider implications of the pandemic on trial outcomes. For example, in a trial of a drug treatment for patients with chronic obstructive pulmonary disease (COPD), the team anticipate that the exacerbation rate may be much lower since participants have been shielding following the UK Government’s guidance [16].

### Re-starting

Although currently recruitment to many trials has been temporarily paused, the NIHR has recently issued guidance on re-starting trials, also relevant for studies funded by UK Research and Innovation (UKRI) [19]. The Restart Framework outlines twelve guiding principles to apply when considering re-starting clinical trials. Trial sponsors, Chief Investigators, trial managers and TMGs are currently considering how to apply the guidance to their trials and whether indeed their trials are still viable to continue. Some trials are considering different approaches to communicating with sites, such as asking key site staff to complete a short survey on whether they are ready to restart. Others have developed a traffic-light system to consider re-start, and some CTUs are considering how best to implement a streamlined approach to their portfolio of trials. There are many uncertainties in restarting clinical trials, and it is too soon to properly assess the issues. Some trial managers have reported conflicting opinions with respect to restart based upon the role of the reporter. For example, Principal Investigators report that the site is ready to restart trial activities, whereas research nurses report lack of capacity to support these studies, since they are continuing to prioritise COVID-19 public health priority studies or being temporarily redeployed to other clinical duties. It is, of course, important that trial teams work in close collaboration with funding bodies, sponsors and NHS partners to ensure a streamlined consistent approach to restart wherever possible. From a longer-term perspective, whilst it is apparent that many studies will require extensions to funding contracts, due to a pause in activity, it is unclear how many studies will require additional funding and research teams welcome a sympathetic approach to this from funders.

### Training

It is a regulatory and ICH-GCP requirement that all staff who work on a clinical trial should be trained to do so [10]. Training should incorporate relevant good clinical practice training and trial-specific training. In many instances, trial-specific training is provided face-to-face, e.g. at a site initiation visit. However, many academic institutions currently have travel and financial restrictions in place, potentially for the foreseeable future, and thus, trial managers are devising alternative methods to provide training. This will also reduce unnecessary footfall in the hospital setting. Whilst the opportunities for utilising technology for training and other meetings existed prior to the pandemic, many research teams continued to utilise traditional approaches, perhaps since this is what they and the site staff they were training were more familiar with. The pandemic has forced research teams to consider new approaches and to implement these quickly, surely a positive to be taken from this international crisis. These include providing remote training by video conferencing facilities, provision of webinars and adding short training videos to trial websites. This provision is relevant to many trials: new trials that are in the set-up phase, ongoing trials where new sites are being set-up, trials where site staff may have changed (either due to COVID-19 or for other reasons) and re-starting trials where re-training may be necessary, dependent upon the length of the pause, the stage of the trial and where significant changes have been made to trial processes as a result of COVID-19. COVID-19 trials have already implemented online training. For example, in the RECOVERY-RS trial [20], members of the research team delivered online site initiation meetings at regular timeslots over several weeks for any site who was able to join. In addition, all training materials were provided via the trial website, rather than paper documentation. Though there are some studies that have evaluated clinical trials training, there is a paucity of evidence with respect to training programmes that may improve site performance [21]. A SWAT embedded in a surgical trial demonstrated that holding online initial contact meetings with sites did not appear to adversely affect set-up times, screening, recruitment or data collection [22]. Developing and implementing new methodologies for training provides opportunities for trial managers, wider research teams and methodologists to evaluate training methods and their potential impact on a range of areas, for example participant recruitment and retention and data quality. The opportunities that web-based interactions enable are great and wide-ranging. Prior to the pandemic, in our experiences, many meetings were either conducted face-to-face (which can often lead to great expense and time, particularly if travel is required) or by tele-conference, which, though more efficient and less costly, has the disadvantage of not having the face-to-face human interaction. Video conferencing, which now features in many of our day-to-day lives, offers some human interaction whilst also being an efficient and less expensive way of meeting.

### Impact on trials in the future

The world still faces a huge amount of uncertainty in light of COVID-19. It is impossible to predict the ongoing impact upon many aspects of everyday life, including how clinical trials may be designed and managed in the future. Processes to comply with regulatory and ethical frameworks have been adapted in order to approve new clinical trials and trial amendments at a speed previously unimagined. For example, the RECOVERY (Randomised Evaluation of COVID-19 Therapy) Trial, which aims to identify treatments that may be beneficial for people hospitalised with suspected or confirmed COVID-19, was set-up in only 9 days—never has this been achieved before [23].

Whilst the pandemic has created many challenges and a monumental effort from trial management teams who have had to implement new procedures, processes and amendments in a time critical period, there are lessons that can be learnt in managing trials in the future. It is well known that clinical trials can be complicated and expensive and therefore efficiencies in trial design and conduct are crucial [24, 25]. We are hopeful that the lessons learnt during these unprecedented times can be carried forward to clinical trials in the future, though clearly a measured approach is required in order to ensure participant safety and adherence with relevant ethical and legislative requirements. Trials should aim to explore, implement and evaluate new technologies and innovations; utilise data routinely available from a range of sources; and at least for the foreseeable future, aim to minimise face-to-face contact with participants wherever possible. The recently launched Good Clinical Trials Collaborative [26], led by Professor Martin Landray at the University of Oxford, which aims to review the principles of randomised clinical trials and provide new guidelines, is welcomed, and UKTMN encourages the clinical trials community to get involved. Additionally, the profile of clinical trials in the general public has been raised during the pandemic. Many members of the public may have participated in a COVID-19 clinical trial or research study themselves, and the urgent need for evidence-based medicine has been highlighted internationally in the media and by senior officials within many governments.

### Sharing best practice

The issues the clinical trial community are faced with are unprecedented. Never before have clinical trial teams had to work in such a way. We have all adapted new ways of working and implemented new processes into clinical trials. To enable efficient, high-quality trials that provide evidence for important health issues in the future, we must come together and work as one. Sharing best practice, lessons learnt and innovative approaches has never been more important. The current clinical trial infrastructure within the UK enables this; the network of 51 UKCRC-registered CTUs play a vital role in the provision of high-quality, relevant methodological expertise in the efficient design and conduct of clinical trials. Our own network, UKTMN, has conducted several online or social media events, to complement its existing forums, to share best practices and innovative ways of working for its 800 members. In addition, a range of other networks and initiatives exist, including the Trials Methodology Research Partnership (TMRP), Trial Forge and the Health Research Board Trials Methodology Research Network (HRB-TMRN) all of which enable continued learning and added value to clinical trials. Many of these groups have developed resources to facilitate shared-learning available on their websites. By working together, alongside sponsors, funding bodies and NHS partners, to learn from one another, the UK is in a unique position to lead the way in designing and conducting high-quality, highly efficient clinical trials.

## Conclusion

The COVID-19 pandemic has resulted in huge changes to the way in which clinical trials are conducted. A cross-cutting theme throughout many of these changes has been the rapid advancement of technology utilisation. All communication has been virtual with trial managers utilising a range of platforms to hold important discussions with trial management groups, independent oversight committees, sites, sponsors, funders and, in some cases, participants. In addition, many trials have moved critical processes such as informed consent and primary outcome data collection online.

Having so rapidly altered many trial processes during earlier stages of the pandemic, trial management teams now need to carefully consider how to approach re-starting their trials. This will require careful planning and a streamlined approach by all stakeholders working harmoniously. There are opportunities for investigators and trial management teams to implement alternative approaches to designing and conducting clinical trials in the future in order to both increase efficiencies and reduce costs, in addition to opportunities for evaluation of these approaches by conducting methodological studies.

## Data Availability

Not applicable.

## Abbreviations

COVID-19: Coronavirus disease
CRN: Clinical Research Network
CTU: Clinical Trials Unit
DMC: Data Monitoring Committee
ED: Emergency Department
EMA: European Medicines Agency
HRB-TMRN: Health Research Board Trials Methodology Research Network
HRA: Health Research Authority
ICH-GCP: International Conference on Harmonisation-Good Clinical Practice
MHRA: Medicines and Healthcare products Regulatory Agency
NIHR: National Institute for Health Research
NHS: National Health Service
TMG: Trial Management Group
TMRP: Trial Methodology Research Partnership
TSC: Trial Steering Committee
UKCRC CTU Network: UK Clinical Research Collaboration Clinical Trials Units Network
URKI: UK Research and Innovation
UKTMN: UK Trial Managers’ Network
WHO: World Health Organization
